# Editorial: Modulation of neuronal excitability by non-neuronal cells in physiological and pathophysiological conditions

**DOI:** 10.3389/fncel.2023.1133445

**Published:** 2023-01-12

**Authors:** Rongqing Chen, Biwen Peng, Peimin Zhu, Yun Wang

**Affiliations:** ^1^Department of Neurobiology, School of Basic Medical Sciences, Southern Medical University, Guangzhou, China; ^2^Hubei Provincial Key Laboratory of Developmentally Originated Disease, Department of Physiology, School of Basic Medical Sciences, Wuhan University, Wuhan, China; ^3^Department of Neurology, Louisiana State University Health Science Center, Shreveport, LA, United States; ^4^Department of Neurology, Institutes of Brain Science, State Key Laboratory of Medical Neurobiology and MOE Frontiers Center for Brain Science, Institute of Biological Science, Zhongshan Hospital, Fudan University, Shanghai, China

**Keywords:** astrocyte, microglia, excitability, hydrogen sulfide, BDNF, polyamine, dexmedetomidine, astrocyte-microglia co-culture

Neuronal excitability is dynamically governed to continuously screen and encode information. For a given neuron in a network, incoming excitatory, inhibitory or modulatory synaptic signals are converged and integrated at the soma to determine its probability of spiking which serves as encoding information for the neuron *per se* as well as output information to its postsynaptic neurons. Neuronal spiking probability is also determined by intrinsic membrane electrical status such as resting membrane potential, membrane conductance, action potential (AP) threshold, and AP profile set up by different types of ion channels. Of note, neurons are surrounded by non-neuronal cells including glial cells which are composed of astrocytes, microglia, oligodendrocytes, NG2 (neuron-glial antigen 2) glia (Jakel and Dimou, 2017), and brain-infiltrated peripheral immune cells (Varvel et al., 2016).

Accumulating evidence suggests that non-neuronal cells play a role in modulating neuronal excitability in the physiological and pathophysiological processes of the brain. For instance, by the actions of potassium channels, e.g., inwardly rectifying potassium channel Kir4.1, and transmitter transporters, e.g., excitatory amino acid transporter-2 (EAAT2) and glutamate aspartate transporter (GLAST, also EAAT1), astrocytes are essentially important in regulating amount of extracellular K^+^ (Coulter and Steinhauser, 2015) and extrasynaptic transmitters (Pajarillo et al., 2019) of neurons and thus influence neuronal excitation. Dysregulation of astrocytic Kir4.1 and glutamate transporters causes neuronal hyper- or hypoexcitability and gives rise to neuropsychiatric disorders such as epilepsy (Nwaobi et al., 2016), depression (Cui et al., 2018), and autism (Pajarillo et al., 2019). As brain-resident macrophage-like cells, microglia actively tune neuronal activity through pruning synapse (Wilton et al., 2019), secreting cytokines (Klapal et al., 2016), contacting with the axon initial segment (AIS) (Cserep et al., 2021), etc. Oligodendrocytes provide axons with myelin sheet enabling fast conduction of action potentials. It is well-described that neural demyelination is associated with multiple sclerosis and some other diseases (Guerrero and Sicotte, 2020). The many ways by which non-neuronal cells influence neuronal excitability and their underlying mechanisms are yet to be researched. Focusing on this, more than 50 authors of 7 articles contributed to this special Research Topic, revealing some interesting novel aspects regarding the modulation of neuronal excitability by glial cells under physiological or pathophysiological conditions.

Three of these articles emphasized the role of astrocytes in regulating brain excitability. By elevating extracellular potassium at a moderate concentration to increase astrocytic but not neuronal volume, Walch et al. investigated the impact of astrocyte swelling to CA1 neuronal excitability. They found that astrocyte swelling increased CA1 neuronal excitability in the form of mixed AMPA/NMDA receptor mediated synaptic transmission. Reversing astrocyte swelling by mannitol dampened the change of neuronal excitability in the presence of elevated extracellular potassium, confirming the contribution of astrocyte swelling to the increased neuronal excitability in elevated extracellular potassium. The authors further demonstrated that the effect of astrocyte swelling on the increment of neuronal excitability mainly resulted from NMDA receptor-mediated large, slow excitatory currents. Polyamines are polycationic molecules which are abundantly stored in astrocytes and upon releasing affect neuronal activity through direct interacting with some ion channels and alter their assembly (Dhara et al., 2020) or ion permeability (Rozov et al., 1998). For instance, spermine is able to produce a rectifying effect of AMPA receptor and some potassium channels by membrane potential-dependent plugging/unplugging the pore of such ion channels (Rozov et al., 1998). In another way, polyamines modulate neuronal activity *via* the availability of astrocyte GABA which can be released from astrocytes and provide tonic inhibition on neurons, but the pathways for polyamines to the production of GABA and the modulation of neuronal excitation are not clear. Kovács et al. addressed the role of astrocytic polyamines on GABA metabolism and epileptic behavior. They revealed that inhibition of the conversion of putrescine to spermidine boosted astrocytic GABA production from putrescine and hence suppresseed neural network excitability and epileptic seizures. It has been suggested that astroglial type 1 cannabinoid receptor (CB1R) mediates synaptic and memory impairments caused by Δ^9^-tetrahydrocannabinol (Δ^9^-THC), the major psychoactive ingredient of marijuana, through COX-2 signaling (Chen et al., 2013). In this topic issue, Cong et al. took use of transgenetic mice with conditional expression of CB1R and revealed that astrocyte COX-2 signaling mediated aversive behavior caused by a high dose of CP 55,940, a synthetic analog of Δ^9^-THC.

Microglia represent a macrophage population in the brain orchestrating a variety of functions including inflammatory response. Hydrogen sulfide (H_2_S) is endogenously synthesized in mammals and is known to regulate a variety of physiological and pathological processes (Kimura, 2021). Zhu et al. (2021) lab synthesized a novel H_2_S donor which is safer than traditional H_2_S donors and is able to release H_2_S effectively in the brain where it exhibits neuroprotective effects against epileptic seizures. To determine its mechanism, they investigated microglial inflammatory profiles associated with this H_2_S donor and found that it reduced seizures by downregulating pro-inflammatory profile while simultaneously increasing anti-inflammatory profile of microglia in pilocarpine-induced status epilepticus mice. Dexmedetomidine (DEX) is a specific and selective alpha-2 adrenoceptor agonist possessing potent anti-neuroinflammatory and neuroprotective properties through the inhibition of pro-inflammatory microglial activation. Wen et al. tested whether such an action of DEX also exists in a neonatal rat model of spinal inflammation and hyperalgesia induced by systemic lipopolysaccharide (LPS) injection. As expected, they found that pretreatment with DEX significantly decreased LPS-induced microglia pro-inflammatory responses and consequentially alleviated LPS-induced mechanical hyperalgesia in neonatal rats. The expression of brain-derived neurotrophic factor (BDNF) in microglia is controversial. Interestingly, by using conditional reporter expression and two-photon imaging, Honey et al. found in the motor cortex that microglia did not express BDNF in sufficient amounts to modulate neuronal dendritic morphology and activity.

Highlighting the *in vitro* astrocyte-microglia co-culture model of inflammation developed two decades ago (Faustmann et al., 2003), Ismail et al. in their mini review summarized the their key findings about glia responsiveness to antiepileptic, psychotropic, neurotrophic, immunomodulatory, and some other brain drugs. They suggest that this unique glia co-culture model of inflammation may be suitable for pharmacological investigations on astrocytes and microglia with future potential. In summary, the articles collected in this special topic present some new aspects of the non-neuronal cells in influencing neuronal activity and their underlying mechanisms.

## Author contributions

All authors listed have made a substantial, direct, and intellectual contribution to the work and approved it for publication.
